# Prioritizing Emergency Medical Conditions for Health Policy Using a Stepwise Treatable-Death Framework: A Nationwide Analysis in Korea, 2018–2022

**DOI:** 10.3390/jcm15207848

**Published:** 2026-10-12

**Authors:** Moon Jeongmin, Chaelin Yoon, Jeong Ho Hyun, Soo Hyun Park, Kyuseok Kim

**Affiliations:** 1 Department of Emergency Medicine, CHA Bundang Medical Center, CHA University School of Medicine, Seongnam 13497, Republic of Korea; a256097@chamc.co.kr (M.J.); hyeun4862@naver.com (J.H.H.); suas11@cha.ac.kr (S.H.P.); 2 National Emergency Medical Center, National Medical Center, Seoul 04564, Republic of Korea; y4lin@nmc.or.kr

**Keywords:** treatable mortality, emergency medical services, health policy, in-hospital mortality, indirect standardization, regional variation, sepsis

## Abstract

**Background/Objectives**: Limited emergency-care resources require priorities based not only on mortality rates or death counts but also on the potential mortality reduction under explicit targets. We applied the age- and sex-standardized domestic short- and mid-term components of a stepwise treatable-death framework across severe emergency conditions in Korea. **Methods**: This retrospective nationwide study used NEDIS aggregate data from 16 regions during 2018–2022. The 20 conditions with the highest numbers of in-hospital deaths were represented as 17 groups by replacing sepsis (A41), bacterial pneumonia (J15), pneumonia of unspecified organism (J18), and pneumonitis due to solids and liquids (J69) with an extended-sepsis composite. Treatable deaths were the positive difference between observed and target-based expected deaths. National rates in 10-year age-group and sex strata were applied to regional distributions. The short-term target assumed that regions with an age- and sex-standardized mortality ratio (SMR) above 1.0 achieved the national reference level (SMR = 1.0), whereas the mid-term target assumed that all regions achieved the lowest regional SMR for the corresponding condition and year. Five-year cumulative estimates were the primary outcome measure. **Results**: Across the 17 groups, summed condition-level patient counts totaled 3,384,529 and in-hospital deaths totaled 357,404 (ratio of summed death counts to summed patient counts, 10.56%). Five-year cumulative short-term estimates were highest for extended sepsis (2387), bronchus and lung cancer (1984), and cardiac arrest (1662). Mid-term estimates were highest for cardiac arrest (21,547), extended sepsis (19,340), and severe trauma (7010). **Conclusions**: After accounting for regional differences in age and sex distributions, extended sepsis remained the condition with the largest short-term and the second-largest mid-term treatable-death burden, supporting its consideration for further policy-priority evaluation. Each treatable-death estimate is an operational policy-scenario estimate rather than an estimate of causally preventable mortality.

## 1. Introduction

Because emergency-care resources are limited, health policy must determine which severe emergency conditions should receive priority [1]. Condition-specific in-hospital mortality rate reflects the risk of death among affected patients, whereas the number of deaths represents the absolute disease burden; however, neither measure alone estimates how many deaths might be reduced if healthcare performance improved to an explicit target. An indicator that incorporates patient volume, observed deaths, and the gap from a target mortality rate may therefore provide a more practical basis for prioritizing emergency-care policy [2,3].

Treatable mortality generally refers to deaths that can be mainly avoided through timely and effective healthcare interventions after disease onset, including secondary prevention and treatment [4]. We adapted this concept to emergency-care policy by proposing a stepwise framework [2]. The present study focuses on its two domestic components: a short-term target assuming that regions with an age- and sex-standardized mortality above the national average achieve the national level, and a mid-term target assuming that all regions achieve the lowest age- and sex-standardized regional mortality level observed in Korea. Treatable death estimated using this framework is a policy-scenario estimate that expresses the difference between observed deaths and deaths expected under a prespecified target mortality level as an absolute number of deaths.

The previous study demonstrated the applicability of the stepwise treatable-death framework to acute myocardial infarction, stroke, and sepsis [2]. However, a limited condition set and a single year of data may not adequately distinguish annual variation or represent policy priorities across a broader range of severe emergency conditions. The domestic short- and mid-term analyses therefore need to be extended to more conditions and multiple years. In addition, differences in regional age and sex distributions could influence comparisons based only on crude mortality rates.

Accordingly, this study aimed to apply the domestic short- and mid-term components of the stepwise treatable-death framework, using age- and sex-standardized estimates, to 17 severe emergency condition groups using nationwide data from 2018 through 2022 and to quantify condition-specific potential reductions in in-hospital deaths under these targets, thereby providing evidence for policy priority setting across severe emergency conditions.

## 2. Materials and Methods

### 2.1. Study Design and Study Period

This was a retrospective nationwide study using data from the National Emergency Department Information System (NEDIS) from 1 January 2018, to 31 December 2022. Analyses were performed by condition, region, and year.

### 2.2. Data Source

We used data from the National Emergency Department Information System (NEDIS) [5]. NEDIS is a nationwide emergency care database that collects standardized information on patients visiting emergency medical institutions in Korea, including demographic characteristics, emergency department (ED) visit and clinical information, diagnoses, ED disposition, and outcomes after hospital admission.

The analysis used information on the diagnoses of the study conditions, location of the treating emergency medical institution, age, sex, and in-hospital mortality. Regions were classified according to the location of the treating emergency medical institution rather than the patient’s residential address.

In-hospital death was defined as death in the ED or death during hospitalization following ED care. The observation period covered the ED visit and subsequent hospitalization, rather than a fixed 30-day follow-up period. Patients who were dead on arrival (DOA) were excluded from the analytic population.

### 2.3. Study Conditions

Study conditions were defined using diagnostic information collected in NEDIS and International Classification of Diseases, 10th Revision (ICD-10) diagnostic codes. Severe trauma was defined using the International Classification of Diseases Injury Severity Score (ICISS), with an ICISS value < 0.9. The initial set comprised the 20 conditions with the highest cumulative numbers of in-hospital deaths during 2018–2022 in the source NEDIS statistics.

For the primary analysis, sepsis (A41), bacterial pneumonia (J15), pneumonia of unspecified organism (J18), and pneumonitis due to solids and liquids (J69) were aggregated within each region and year and combined into a composite category termed extended sepsis, resulting in 17 final condition groups. This operational composite was intended to capture the broader burden of severe infection-related emergency conditions that may be fragmented across diagnostic categories in administrative data and should not be interpreted as a clinical Sepsis-3 definition. For the condition-level analysis, each patient was assigned to a single study diagnosis in the source dataset; therefore, the four infection-related categories were mutually exclusive and no patient was counted more than once within the extended-sepsis composite. The four component diagnoses were not included separately in the primary analysis. The final study conditions and their diagnostic codes or criteria are presented in Table 1.

### 2.4. Study Regions

Regional analyses were conducted across 16 units: Seoul, Busan, Daegu, Incheon, Gwangju, Daejeon, Ulsan, Gyeonggi, Gangwon, Chungbuk, Chungnam-Sejong, Jeonbuk, Jeonnam, Gyeongbuk, Gyeongnam, and Jeju. Regional classification was based on the location of the treating emergency medical institution rather than the patient’s residence.

### 2.5. Age- and Sex-Standardized Mortality Ratios

To account for regional differences in age and sex distributions, standardized mortality ratios (SMRs) were calculated using indirect standardization. Age was categorized into 10-year groups and jointly stratified with sex.

Among the overall study population in the 17 final condition groups, 29 records had unknown age information, including 8 in-hospital deaths. These records were retained in the overall study population but were excluded from the age- and sex-adjusted SMR and treatable-death analyses because they could not be assigned to a 10-year age-by-sex stratum. No additional records were excluded because of missing sex or treating-institution region information.

For each condition and year, national in-hospital mortality rates were calculated for each 10-year age group and sex stratum using data pooled across all 16 regions. The expected number of deaths for each region was then calculated by applying these national stratum-specific mortality rates to the corresponding regional patient population.

For each condition and year, the expected number of deaths in region r was calculated as follows:

$$E_{r}=\sum_{a,s}N_{ras}\times m_{as}$$

where E_r_ is the expected number of deaths in region r, N_ras_ is the number of patients in region r within age group a and sex s, and m_as_ is the corresponding national in-hospital mortality rate. All quantities were calculated within the same condition and year.

The SMR for each region was calculated as:

$${SMR}_{r}=\frac{O_{r}}{E_{r}}$$

where O_r_ is the observed number of in-hospital deaths in region r. An SMR > 1.0 indicated that the observed number of deaths exceeded the number expected based on the national age- and sex-specific mortality rates for the corresponding condition and year.

### 2.6. 95% Confidence Intervals for SMRs and Recurrent Elevation

For each condition-, region-, and year-specific SMR, the 95% confidence interval (CI) was calculated using the exact Poisson method, assuming that the observed number of deaths followed a Poisson distribution. This approach derives confidence limits from the Poisson distribution of the observed death count without requiring a normal approximation and is an established exact approach for inference on SMRs [6]. An SMR > 1.0 was described as mortality above the national reference level, whereas a lower bound of the exact Poisson 95% CI > 1.0 was considered to indicate a statistically significant elevation above the national reference. Recurrent elevation was defined as meeting the respective criterion in at least 3 of the 5 study years from 2018 through 2022.

### 2.7. Definition and Estimation of Treatable Deaths

Treatable deaths were defined as the difference between the observed number of deaths and the number expected under a prespecified target mortality level. Each treatable-death estimate is an operational policy-scenario estimate rather than an estimate of causally preventable mortality. To account for regional differences in patient age and sex distributions, the age- and sex-standardized expected numbers of deaths and SMRs derived above were used. When the observed number of deaths was lower than the number expected under the corresponding target, the treatable-death estimate was set to zero.

#### 2.7.1. Short-Term Target

The short-term target represented a scenario in which the age- and sex-standardized mortality level of each region reached the national level for the same condition and year. Because E for each region was calculated by applying national age- and sex-specific in-hospital mortality rates for the same condition and year to the regional patient distribution, the national reference level corresponded to an SMR of 1.0.

$${TD}_{r}^{short}=max\left[0,O_{r}-E_{r}\right]$$

Regions with observed deaths at or below the expected number contributed zero treatable deaths. National short-term treatable deaths for each condition and year were calculated by summing the regional estimates across all 16 regions.

$${TD}^{short}=\sum_{r}{TD}_{r}^{short}$$

Thus, the short-term estimate represented the estimated number of deaths that could be reduced if the age- and sex-standardized mortality level of each region improved to the national level for the same condition and year.

#### 2.7.2. Mid-Term Target

The mid-term target represented a scenario in which all regions achieved the mortality level of the region with the lowest age- and sex-standardized SMR among the 16 regions for the same condition and year.

$${SMR}^{min}=\underset{r}{min}\left({SMR}_{r}\right)$$

For each region, the mid-term target expected number of deaths was calculated by multiplying its age- and sex-standardized expected number of deaths by the lowest regional SMR for the corresponding condition and year.

$$E_{r}^{target}=E_{r}\times {SMR}^{min}$$

The regional contribution to the mid-term treatable-death estimate was calculated as follows:

$${TD}_{r}^{mid}=max\left[0,O_{r}-E_{r}^{target}\right]$$

National mid-term treatable deaths for each condition and year were calculated by summing the regional contributions across all 16 regions.

$${TD}^{mid}=\sum_{r}{TD}_{r}^{mid}$$

Thus, the mid-term estimate represented the estimated number of deaths that could be reduced if the age- and sex-standardized mortality level of each region improved to the lowest regional level observed nationally for the same condition and year.

#### 2.7.3. Five-Year Cumulative and Mean Annual Treatable Deaths

**Figure 1:**
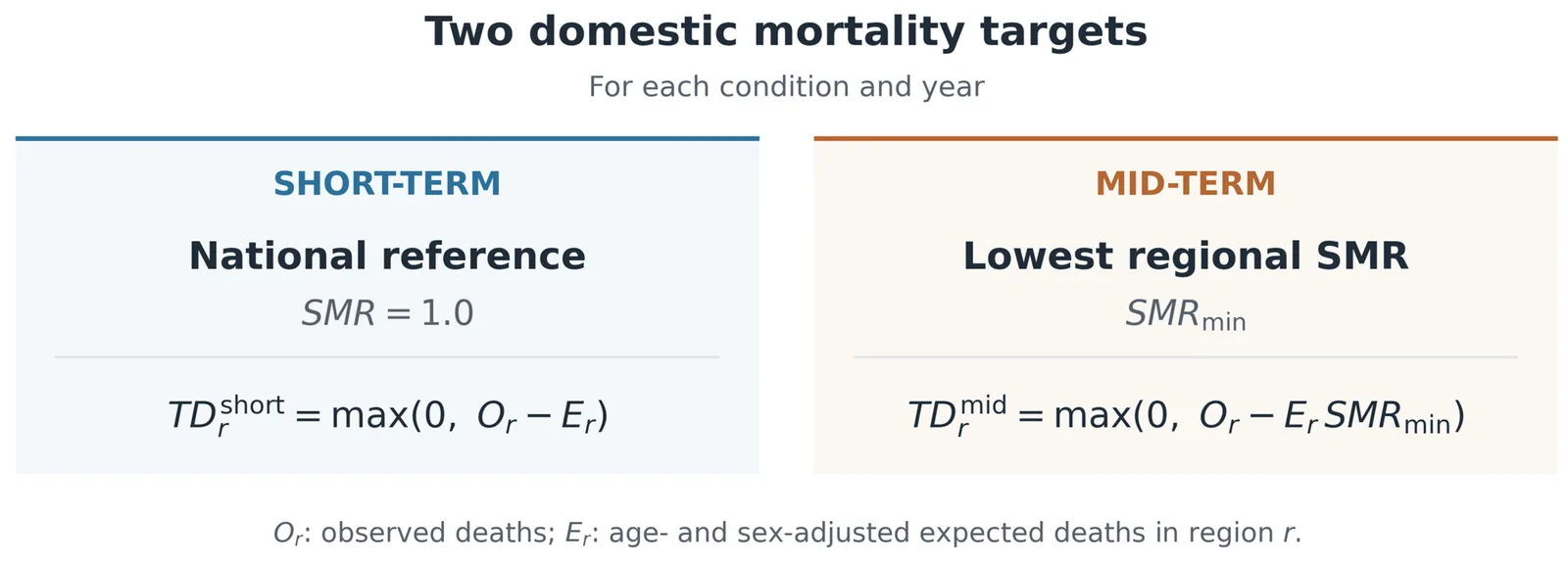
Stepwise framework for estimating treatable deaths under short- and mid-term targets. The two targets represent complementary domestic policy scenarios rather than a single sequential benchmark.

Short- and mid-term treatable deaths were estimated separately for each year from 2018 through 2022. Five-year cumulative treatable deaths for each condition were calculated by summing the annual estimates, and the mean annual estimate was calculated by dividing the five-year cumulative value by 5. Condition rankings were determined using the full-precision five-year cumulative estimates before rounding for presentation. The analytical framework is shown in Figure 1.

### 2.8. Supplementary Analyses

To examine the influence of age and sex standardization, short- and mid-term treatable deaths were additionally calculated without age or sex standardization using the same analytic population as the adjusted analyses. For each condition and year, the crude short-term target was the national crude in-hospital mortality rate, and the crude mid-term target was the lowest regional crude in-hospital mortality rate. Annual and five-year cumulative crude estimates for the 17 primary-analysis condition groups are presented in Supplementary Tables S2 and S3. The original 20 individual conditions were also summarized separately using the full source population, including records with unknown age because no age or sex standardization was applied, with the four infection-related diagnoses retained individually and the extended-sepsis composite excluded (Supplementary Table S1). This table preserves component-level diagnostic information and was not used to determine the primary 17-group rankings.

Two sensitivity analyses used alternative mid-term benchmarks: pooled SMRs of the three regions with the lowest SMRs within each condition and year, and of the three regions with the lowest five-year SMRs. Five-year regional SMRs were calculated as cumulative observed deaths divided by cumulative expected deaths. Each benchmark was the selected regions’ summed observed deaths divided by summed expected deaths over the corresponding period; the five-year benchmark was applied unchanged across years. Using the original condition- and year-specific age- and sex-standardized expected deaths, each region-year estimate was calculated as observed deaths minus the product of expected deaths and the benchmark, truncated at zero before summation (Supplementary Table S5).

To assess the influence of the extended-sepsis grouping, we performed a sensitivity analysis in which the extended-sepsis composite was removed and A41, J15, J18, and J69 were entered separately, while the other 16 primary-analysis conditions were retained, yielding 20 condition groups. The same age- and sex-adjusted (10-year age groups) indirect standardization and treatable-death estimation procedures used in the primary analysis were applied to all 20 conditions. We recalculated five-year cumulative short- and mid-term treatable-death rankings and, for each condition and region, counted the number of years from 2018 through 2022 with an annual SMR > 1 and with the lower bound of the exact Poisson 95% CI > 1. Recurrent elevation was defined as meeting the corresponding criterion in at least 3 of 5 years. Mid-term estimates using the annually selected pooled lowest-three benchmark were also calculated for these 20 condition groups (Supplementary Table S6).

### 2.9. Statistical Analysis

Patient counts, in-hospital deaths, age- and sex-standardized expected deaths, SMRs, and 95% CIs were calculated by condition, region, and year, and these measures were used to estimate short- and mid-term treatable deaths. All SMR analyses and the primary treatable-death analyses used indirect standardization with simultaneous adjustment for sex and 10-year age groups. Expected deaths, SMRs, confidence intervals, treatable-death estimates, and condition rankings were calculated using full-precision values, with rounding applied only for presentation. All analyses were performed using Python (version 3.14.2; Python Software Foundation, Wilmington, DE, USA; https://www.python.org/). Data processing and numerical calculations were conducted using pandas (version 3.0.5; https://pandas.pydata.org/) and NumPy (version 2.5.2; https://numpy.org/), and exact Poisson 95% confidence intervals and Spearman rank correlation coefficients were calculated using SciPy (version 1.18.1; https://scipy.org/).

Rank stability of condition-specific five-year cumulative mid-term treatable-death estimates among the 17 primary-analysis condition groups was assessed using Spearman’s rank correlation coefficient between the primary single-lowest and annually selected pooled lowest-three benchmark rankings. Spearman’s coefficient was used because the objective was to evaluate preservation of the relative ordering of conditions despite changes in the absolute estimates. We additionally calculated Top-5 overlap, defined as the number of conditions common to the five highest-ranked conditions under both benchmarks, irrespective of their order within the Top 5.

### 2.10. Artificial Intelligence Use

A generative artificial intelligence tool, ChatGPT using the GPT-5.6 Sol model (OpenAI, San Francisco, CA, USA), was used solely for language editing, grammatical correction, and refinement of English expression. The authors reviewed and approved all AI-assisted revisions and remain fully responsible for the final content.

## 3. Results

### 3.1. Study Population

Across the 17 primary-analysis condition groups, summed condition-level patient counts totaled 3,384,529 and in-hospital deaths totaled 357,404, corresponding to a ratio of summed death counts to summed patient counts of 10.56% (Table 1). Extended sepsis accounted for 577,907 patients and 74,284 in-hospital deaths, with a mortality rate of 12.85%. It had the second-largest patient count after intracranial injury (S06; 768,748) and the second-largest number of in-hospital deaths after cardiac arrest (I46; 90,213).

### 3.2. Age- and Sex-Standardized Short-Term Treatable Deaths

**Figure 2:**
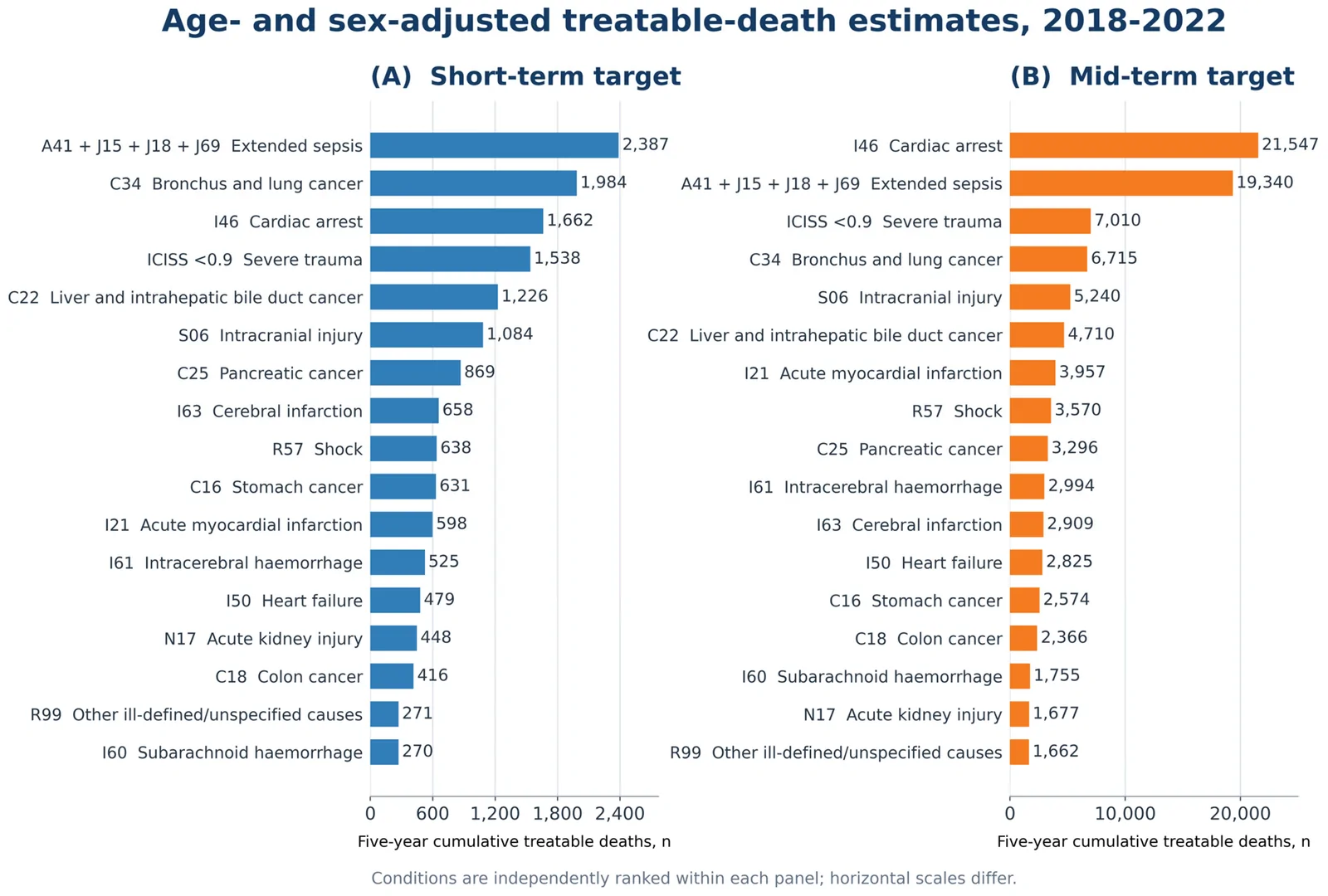
Five-year cumulative age- and sex-adjusted short- (**A**) and mid-term (**B**) treatable-death estimates for the 17 primary-analysis condition groups, 2018–2022. Conditions are ordered independently in descending order of the estimate within each panel, and the two panels use different horizontal scales.

Under the age- and sex-standardized short-term target, five-year cumulative treatable deaths were highest for extended sepsis (2387), followed by bronchus and lung cancer (C34; 1984), cardiac arrest (I46; 1662), severe trauma (ICISS < 0.9; 1538), liver and intrahepatic bile duct cancer (C22; 1226), and intracranial injury (S06; 1084) (Table 2). The corresponding mean annual estimates were 477.3, 396.8, 332.4, 307.6, 245.1, and 216.8, respectively. Table 2 also presents the annual age- and sex-adjusted short-term estimates from 2018 through 2022. The five-year cumulative short- and mid-term estimates are compared visually in Figure 2; the corresponding crude short-term estimates are presented in Supplementary Table S2.

### 3.3. Age- and Sex-Standardized Mid-Term Treatable Deaths

Under the age- and sex-standardized mid-term target, five-year cumulative treatable deaths were highest for cardiac arrest (I46; 21,547), followed by extended sepsis (19,340), severe trauma (ICISS < 0.9; 7010), bronchus and lung cancer (C34; 6715), intracranial injury (S06; 5240), and liver and intrahepatic bile duct cancer (C22; 4710) (Table 3). The corresponding mean annual estimates were 4309.4, 3868.1, 1402.1, 1343.1, 1047.9, and 942.1, respectively. Table 3 also presents the annual age- and sex-standardized mid-term estimates from 2018 through 2022. The five-year cumulative short- and mid-term estimates are compared visually in Figure 2; the corresponding crude mid-term estimates are presented in Supplementary Table S3.

Both alternative benchmarks retained the same six leading condition groups as the primary mid-term analysis (Supplementary Table S5). Extended sepsis ranked first, with five-year cumulative estimates of 13,035 using annually selected three-region benchmarks and 13,314 using the five-year pooled benchmark; cardiac arrest ranked second (11,047 and 11,294, respectively).

When the mid-term benchmark was changed from the single region with the lowest adjusted SMR to a pooled benchmark based on the three regions with the lowest adjusted SMRs for each condition and year, the absolute five-year mid-term treatable-death estimate decreased for all 17 conditions. However, rankings remained highly concordant (Spearman ρ = 0.978, *p* < 0.001), and all five conditions in the primary-analysis Top 5 remained in the Top 5 under the sensitivity benchmark (Supplementary Table S5).

Extended sepsis remained the highest-ranked short-term condition in both 2018–2019 and 2020–2022 and remained among the two highest-ranked conditions under the mid-term scenario in both periods.

### 3.4. Age- and Sex-Standardized Regional SMR Analysis

Using the descriptive criterion of an annual SMR greater than 1.0, recurrent descriptive elevation in at least three of the five study years was most widespread for liver and intrahepatic bile duct cancer (12 of 16 regions), followed by pancreatic cancer and bronchus and lung cancer (11 regions each), and extended sepsis and acute kidney injury (10 regions each). Using the CI-based criterion of a lower exact Poisson 95% CI limit greater than 1.0, recurrent CI-based elevation was observed in seven regions for bronchus and lung cancer, six for pancreatic cancer, four each for liver and intrahepatic bile duct cancer and severe trauma, and three each for extended sepsis, stomach cancer, and intracranial injury. No region showed recurrent CI-based elevation for subarachnoid haemorrhage or other ill-defined and unspecified causes of mortality. Full results are presented in Supplementary Table S4 and Supplementary Figure S1. This indicates that age- and sex-standardized SMR elevation signals for these indicators recurred over multiple years rather than being confined to a single year. These elevations may reflect residual case-mix differences and should not be interpreted as direct measures of emergency-care quality.

### 3.5. Supplementary Analyses

The original 20 individual-condition crude analysis, in which the four infection-related diagnoses were retained separately and the extended-sepsis composite was excluded, is reported in Supplementary Table S1. The leading conditions in the crude analyses of the 17 primary-analysis groups were broadly consistent with those in the age- and sex-adjusted primary analysis. Adjustment changed the ordering of several adjacent conditions, including liver and intrahepatic bile duct cancer versus intracranial injury in the short-term analysis and severe trauma versus bronchus and lung cancer in the mid-term analysis (Supplementary Tables S2 and S3).

In the 20-condition sensitivity analysis, in which extended sepsis was replaced by A41, J15, J18, and J69, J18 had a five-year cumulative short-term treatable-death estimate of 1990.0, ranking first, and a mid-term estimate of 12,955.2 under the single-lowest regional benchmark, ranking second. A41 ranked seventh and fifth, respectively; J69 ranked 13th and 11th; and J15 ranked 18th and 17th. Across all 20 conditions, recurrent descriptive SMR elevation (SMR > 1 in at least 3 of 5 years) was observed in 12 regions for C22, 11 each for C34 and C25, and 10 each for A41 and N17, among others. Recurrent statistically significant elevation (exact Poisson 95% CI lower bound > 1 in at least 3 of 5 years) was observed in 7 regions for C34, 6 for C25, and 4 each for J18, severe trauma, and C22 (Supplementary Table S6).

Among the four component conditions of extended sepsis, recurrent descriptive elevation was observed in 10 regions for A41, 9 for J15, 8 for J18, and 8 for J69; recurrent statistically significant elevation was observed in 2, 0, 4, and 3 regions, respectively. These findings show that recurrent regional mortality elevation remained evident for several component conditions after disaggregation while also demonstrating heterogeneity across the components (Supplementary Table S6).

## 4. Discussion

This nationwide study used five years of data to estimate potential reductions in in-hospital deaths across 17 severe emergency condition groups under the age- and sex-standardized domestic short- and mid-term targets. Under the short-term target, extended sepsis had the largest five-year cumulative estimate (2387), followed by bronchus and lung cancer (C34; 1984). Under the mid-term target, cardiac arrest (I46) had the largest estimate (21,547), followed by extended sepsis (19,340). Substantial regional differences remained after adjustment for age and sex, suggesting that factors beyond regional age and sex composition may also have contributed to the observed variation.

Extended sepsis ranked first in the short-term scenario and second in the mid-term scenario for treatable deaths, suggesting that its policy relevance arises not simply from a high mortality rate but from the combination of a large affected population and a substantial mortality gap from domestic benchmarks. This distinction is important for priority setting because mortality rates or absolute death counts considered in isolation may not identify conditions in which improvements in healthcare performance could yield the greatest reduction in deaths.

The disaggregated analysis supports further policy evaluation of infection-related conditions, particularly J18, while demonstrating that the component diagnoses differed in their estimated burden and patterns of recurrent regional SMR elevation.

Concrete examples demonstrate that disease-specific policies can alter both care delivery and clinical outcomes. In cancer, national cancer-control programs have expanded organized screening, prevention, data infrastructure, and coordinated treatment pathways, although their effect on mortality varies by program and population; for example, Korea’s National Cancer Control Plan has progressively expanded prevention, screening, treatment, and survivorship infrastructure, while international analyses suggest that dedicated cancer-control plans are associated with improvements in several population-level cancer-control indicators [7,8]. In dementia, national dementia plans have been established across G7, European, and other high-income countries, with coordinated strategies encompassing prevention, diagnosis, long-term care, caregiver support, research, and information systems [9,10]. Implementation of the English National Dementia Strategy was associated with a significant increase in recorded dementia diagnoses and prescriptions of antidementia medications, illustrating how national policy can increase case recognition and access to treatment [11]. For acute myocardial infarction, regional and national STEMI systems of care in Europe and North America have integrated emergency medical services, designated PCI-capable hospitals, interhospital transfer protocols, and quality monitoring to improve timely reperfusion and clinical outcomes [12]. Regionalized STEMI systems linking emergency medical services with PCI-capable hospitals have shortened reperfusion times and, in some programs, reduced in-hospital and long-term mortality [12,13]. In Korea, the Second Comprehensive Plan for Cardiovascular and Cerebrovascular Disease Management (2023–2027) emphasizes timely treatment and coordinated care networks [14]. Stroke has likewise received extensive policy and organizational support through national stroke plans, specialized stroke units, organized acute-care pathways, and international initiatives such as the Stroke Action Plan for Europe [15]. Organized stroke-unit care has consistently reduced mortality, dependency, and institutionalization compared with conventional ward care [16]. Regionalized trauma systems have likewise been associated with lower injury mortality, and in Korea the development of regional trauma centers was accompanied by a decrease in the national preventable trauma death rate from 30.5% in 2015 to 19.9% in 2017 [17,18]. These examples suggest that disease-specific policy can translate into measurable improvements when it establishes defined care pathways, dedicated infrastructure, timely referral or treatment, and continuous outcome monitoring.

Sepsis, by comparison, may represent a mismatch between disease burden and disease-specific policy prioritization. Despite an estimated 48.9 million cases and 11.0 million sepsis-related deaths worldwide in 2017 [19], and the World Health Assembly’s explicit call for national strategies to improve sepsis prevention, recognition, diagnosis, and treatment [20], disease-specific national systems for sepsis remain less consistently developed than those for cancer, dementia, acute cardiovascular and cerebrovascular diseases, or trauma. Given the recommendations of the World Health Assembly and the substantial global and Korean burden of sepsis [19,20,21], a more explicit national approach should be implemented. Sepsis presents a different policy challenge because it is not defined by a single anatomical disease process or a single diagnostic test and may initially manifest with nonspecific or subtle findings. A sepsis-specific national strategy would therefore need to combine clinical recognition with system organization rather than rely on a single screening criterion. Potential components include standardized surveillance and administrative definitions, structured reassessment of patients with suspected infection and evolving organ dysfunction, predefined escalation pathways, timely antimicrobial and source-control processes, regional referral pathways for patients requiring critical care, and nationally reported quality indicators. Public and professional education, regular audit and feedback, and benchmarking of hospital and regional outcomes could provide additional mechanisms for system improvement. Such measures would parallel the organized pathways established for STEMI, stroke, trauma, cancer, and dementia, while accounting for the diagnostic heterogeneity and time-sensitive nature of sepsis. The treatable-death framework may therefore provide a quantitative approach for identifying conditions, such as sepsis, for further policy evaluation when the potential mortality-reduction burden may be disproportionate to their current visibility within disease-specific health policy.

Importantly, the overall prioritization pattern was largely preserved after age and sex standardization, although the ordering of several adjacent conditions changed. This suggests that the large treatable-death burden of extended sepsis was not primarily attributable to regional differences in demographic composition.

Although the order and magnitude of the estimates differed, the same six condition groups appeared in the top six under both age- and sex-standardized domestic targets, indicating substantial overlap in the conditions identified for priority review. Within this pattern, the large estimated mortality-reduction burden for extended sepsis supports its consideration alongside other major severe emergency conditions. However, each treatable-death estimate is an operational policy-scenario estimate rather than an estimate of causally preventable mortality; clinical actionability, feasibility, and resource requirements should be evaluated before it is used to guide policy decisions.

Recurrent regional SMR elevations were observed for several conditions, including extended sepsis, suggesting persistent geographic variation after age and sex standardization. Because adjustment was limited to age and sex, these differences do not establish poorer emergency-care performance or an achievable improvement through care alone.

The two domestic targets address complementary policy questions. Short-term estimates indicate where reducing regional mortality gaps may yield meaningful gains, whereas mid-term estimates indicate the potential associated with approaching the lowest observed domestic SMR and can guide investigation of the factors underlying lower mortality. The framework is therefore best used as a screening tool that connects potential mortality reduction with different levels of domestic policy action.

This study has several strengths. First, it used nationwide data spanning five consecutive years, thereby reducing the influence of fluctuations in any single year. Second, a transparent and reproducible age- and sex-standardized target-based method was applied across a broad range of severe emergency conditions, allowing patient volume and the mortality gap from an explicit target to be expressed as an absolute number of deaths. Third, the age- and sex-standardized SMR analysis provided a complementary assessment of regional variation, persistence, and statistical uncertainty.

### Limitations

Several limitations should be considered. First, the primary treatable-death analyses used condition-, region-, and year-level aggregate data. Although the analyses adjusted for age and sex, patient-level information on comorbidities, clinical severity, transfer status, or hospital characteristics was unavailable. Totals across condition groups were sums of mutually exclusive condition-level counts. Differences in other aspects of case mix and care settings could not be adjusted for, and the aggregate findings should not be interpreted as individual-level associations or causal effects. Because the outcome was restricted to in-hospital mortality, deaths after discharge were not captured. Patient-level linkage was also unavailable to trace care pathways across interhospital transfers. Regional differences in length of stay and discharge or transfer practices may therefore have influenced comparisons based on in-hospital mortality. Accordingly, the treatable-death estimates represent differences between observed deaths and target-based expected deaths under specified scenarios, rather than numbers of deaths demonstrated to have been causally preventable through healthcare intervention.

Second, estimates were inherently dependent on the selected targets. Although both three-region sensitivity analyses retained the same six leading condition groups, the lower estimates indicate sensitivity of the absolute magnitude to benchmark selection. In particular, the lowest annual regional SMR used for the mid-term target may have been influenced by small patient volumes, random variation, coding practices, and unmeasured case mix. No reliability adjustment or shrinkage procedure was applied, and the lowest observed regional level may not represent a stable or reproducibly attainable benchmark. Moreover, indirectly standardized SMRs for different populations do not necessarily have identical effective standard populations and therefore require caution when directly compared [22]. The mid-term findings should consequently be interpreted as exploratory policy-scenario estimates based on the lowest observed domestic SMR rather than as definitive estimates of achievable mortality reduction. Although the extended-sepsis composite was used to capture a broader burden of severe infection-related emergency conditions, it is an operational administrative construct rather than a clinical Sepsis-3 definition. Accordingly, some patients classified under pneumonia- or aspiration-related diagnostic categories may not have met clinical criteria for sepsis, and the composite should therefore be interpreted as a broader infection-related burden rather than as a precise estimate of clinically defined sepsis.

Third, the source data grouped shock as a single condition and did not provide its etiologic subtypes. In a large population-based linked cohort of adults with emergency-medical-services-treated nontraumatic shock, cardiogenic, septic, hypovolaemic, and other causes accounted for 37.6%, 36.6%, 10.0%, and 15.8% of 30-day deaths, respectively, when calculated from the reported death counts [23]. These external proportions cannot be applied directly to the NEDIS population because the care setting, case definition, and mortality window differ, but they indicate that infection-related shock may account for a substantial proportion of shock deaths. Septic shock is defined clinically as a subset of sepsis [24]; therefore, future studies using etiologic or patient-level information should examine whether deaths due to septic shock should be incorporated into the extended-sepsis burden. The present data did not allow the number of septic-shock deaths within the shock category or the effect of reclassifying them on the relative rankings of shock and extended sepsis to be quantified.

Fourth, we did not include a long-term international target in this study because comparable external benchmarks were unavailable across all study conditions using consistent diagnostic definitions, patient populations, mortality time windows, adjustment methods, and reference years. Applying heterogeneous benchmarks to selected conditions could have produced apparent differences driven by data-source incompatibility rather than true performance gaps. We therefore confined the priority-ranking framework to the two internally comparable domestic targets.

Finally, the study period included the COVID-19 pandemic. During 2020–2022, Korea had a relatively low cumulative reported COVID-19 mortality rate of fewer than 70 deaths per 100,000 population, compared with an OECD average of 225 [25]. Nevertheless, pandemic-related changes in emergency department utilization and mortality among patients with major emergency conditions in Korea [26] may have influenced the present estimates.

## 5. Conclusions

After accounting for regional differences in age and sex distributions, extended sepsis had the largest short-term and second-largest mid-term target-based mortality-reduction estimate. These findings identify severe infection-related conditions as an important area for further policy-priority evaluation, while decisions regarding national resource allocation should additionally consider intervention effectiveness, feasibility, costs, and competing healthcare priorities. Each treatable-death estimate is an operational policy-scenario estimate rather than an estimate of causally preventable mortality. Future studies using patient-level risk-adjusted data are needed to evaluate the feasibility and expected effects of condition-specific interventions.

## Figures and Tables

**Table 1:** Primary-analysis condition groups and cumulative in-hospital outcomes, 2018–2022.

Code or Criterion	Study Condition	Patients, n	In-Hospital Deaths, n	Mortality, %
A41 + J15 + J18 + J69	Extended sepsis	577,907	74,284	12.85
C16	Stomach cancer	81,020	7951	9.81
C18	Colon cancer	71,652	5566	7.77
C22	Liver and intrahepatic bile duct cancer	125,268	15,904	12.70
C25	Pancreatic cancer	78,966	8391	10.63
C34	Bronchus and lung cancer	147,261	23,149	15.72
I21	Acute myocardial infarction	150,783	10,661	7.07
I46	Cardiac arrest	106,564	90,213	84.66
I50	Heart failure	132,791	8896	6.70
I60	Subarachnoid haemorrhage	41,650	6344	15.23
I61	Intracerebral haemorrhage	77,166	10,501	13.61
I63	Cerebral infarction	332,018	10,398	3.13
ICISS < 0.9	Severe trauma	559,635	28,906	5.17
N17	Acute kidney injury	74,364	6353	8.54
R57	Shock	31,028	10,337	33.32
R99	Other ill-defined and unspecified causes of mortality	27,708	26,557	95.85
S06	Intracranial injury	768,748	12,993	1.69
**—**	**Total**	**3,384,529**	**357,404**	**10.56**

Note. Extended sepsis was calculated as the region- and year-specific sum of A41 (other sepsis), J15 (bacterial pneumonia, not elsewhere classified), J18 (pneumonia, organism unspecified), and J69 (pneumonitis due to solids and liquids); these component conditions are not presented separately in the primary analysis. Severe trauma was defined as an ICISS below 0.9. Totals are sums of mutually exclusive condition-level counts (in bold); the same case was not counted in more than one study diagnostic category. ICD-10, International Classification of Diseases, 10th Revision; ICISS, International Classification of Diseases Injury Severity Score.

**Table 2:** Annual and five-year cumulative age- and sex-standardized short-term treatable-death estimates and rankings for the 17 primary-analysis condition groups, 2018–2022.

Rank	Code or Criterion	Study Condition	2018	2019	2020	2021	2022	5-Year Cumulative	Mean Annual
1	A41 + J15 + J18 + J69	Extended sepsis	462	512	466	463	483	2387	477.3
2	C34	Bronchus and lung cancer	448	404	423	369	339	1984	396.8
3	I46	Cardiac arrest	294	374	389	318	287	1662	332.4
4	ICISS < 0.9	Severe trauma	316	326	280	313	303	1538	307.6
5	C22	Liver and intrahepatic bile duct cancer	267	298	243	270	147	1226	245.1
6	S06	Intracranial injury	218	222	226	222	196	1084	216.8
7	C25	Pancreatic cancer	150	206	181	164	167	869	173.8
8	I63	Cerebral infarction	127	121	120	136	155	658	131.5
9	R57	Shock	104	136	120	124	155	638	127.6
10	C16	Stomach cancer	130	142	119	155	85	631	126.2
11	I21	Acute myocardial infarction	112	145	89	134	118	598	119.6
12	I61	Intracerebral haemorrhage	123	96	87	106	113	525	105.0
13	I50	Heart failure	82	94	97	110	96	479	95.9
14	N17	Acute kidney injury	95	72	96	107	78	448	89.6
15	C18	Colon cancer	91	88	55	84	98	416	83.2
16	R99	Other ill-defined and unspecified causes of mortality	77	67	40	41	46	271	54.1
17	I60	Subarachnoid haemorrhage	50	60	65	53	42	270	54.1

**Table 3:** Annual and five-year cumulative age- and sex-standardized mid-term treatable-death estimates and rankings for the 17 primary-analysis condition groups, 2018–2022.

Rank	Code or Criterion	Study Condition	2018	2019	2020	2021	2022	5-Year Cumulative	Mean Annual
1	I46	Cardiac arrest	4055	4636	5087	4286	3484	21,547	4309.4
2	A41 + J15 + J18 + J69	Extended sepsis	2687	3171	4747	4480	4255	19,340	3868.1
3	ICISS < 0.9	Severe trauma	1532	1911	930	1349	1288	7010	1402.1
4	C34	Bronchus and lung cancer	1105	1745	1104	1421	1341	6715	1343.1
5	S06	Intracranial injury	898	1277	1088	1053	923	5240	1047.9
6	C22	Liver and intrahepatic bile duct cancer	804	750	1065	1286	805	4710	942.1
7	I21	Acute myocardial infarction	743	752	543	1095	824	3957	791.3
8	R57	Shock	579	824	682	712	772	3570	713.9
9	C25	Pancreatic cancer	603	794	782	662	454	3296	659.1
10	I61	Intracerebral haemorrhage	737	488	740	515	514	2994	598.8
11	I63	Cerebral infarction	543	562	591	547	666	2909	581.8
12	I50	Heart failure	655	594	522	529	525	2825	565.1
13	C16	Stomach cancer	336	562	615	632	429	2574	514.9
14	C18	Colon cancer	261	471	314	549	771	2366	473.3
15	I60	Subarachnoid haemorrhage	303	313	258	400	482	1755	351.0
16	N17	Acute kidney injury	350	363	401	356	207	1677	335.4
17	R99	Other ill-defined and unspecified causes of mortality	581	238	280	213	350	1662	332.4

## Data Availability

The NEDIS-derived data analyzed in this study were provided separately by the National Emergency Medical Center of the National Medical Center, Republic of Korea, for the approved study. The data are not publicly available, and the authors are not authorized to redistribute them because access is restricted by the data provider and the conditions of Institutional Review Board approval. Access, if available, would require a separate request to and approval from the National Emergency Medical Center. The derived results needed to interpret the findings are presented in the article and Supplementary Materials.

## Supplementary Materials

The following supporting information can be downloaded at: https://www.mdpi.com/article/10.3390/jcm15207848/s1, Supplementary Table S1: Original 20 individual-condition analyses, 2018–2022; Supplementary Table S2: Annual and five-year cumulative crude short-term treatable-death estimates and rankings for the 17 primary-analysis condition groups, 2018–2022; Supplementary Table S3: Annual and five-year cumulative crude mid-term treatable-death estimates and rankings for the 17 primary-analysis condition groups, 2018–2022; Supplementary Table S4: Recurrent descriptive and CI-based age- and sex-adjusted regional SMR elevations by condition, 2018–2022; Supplementary Table S5: Sensitivity analyses of five-year cumulative age- and sex-standardized mid-term treatable-death estimates using alternative regional benchmarks, 2018–2022; Supplementary Table S6: Sensitivity analysis after disaggregating extended sepsis into its four component conditions; Supplementary Figure S1: Heatmaps of age- and sex-adjusted regional SMR elevations by condition and anonymized region, 2018–2022.
