# Supplementary material for: Prioritizing Emergency Medical Conditions for Health Policy Using a Stepwise Treatable-Death Framework: A Nationwide Analysis in Korea, 2018–2022
Source: J Clin Med. 2026 Oct 12;15(20):7848. doi: 10.3390/jcm15207848 (PMC13705308; doi:10.3390/jcm15207848)

Supplementary Materials

Supplementary Table S1. Original 20 individual-condition analyses, 2018–2022

Code or criterion	Condition	Patients, n	Deaths, n	Mortality, %	Short cumulative	Short mean annual	Short rank	Mid cumulative	Mid mean annual	Mid rank
J18	Pneumonia, unspecified organism	411,513	43,547	10.58	2,447	489	1	12,290	2,458	2
C34	Bronchus and lung cancer	147,261	23,149	15.72	2,247	449	2	6,573	1,315	3
I46	Cardiac arrest	106,564	90,213	84.66	1,669	334	3	22,002	4,400	1
ICISS <0.9	Severe trauma	559,635	28,906	5.17	1,508	302	4	6,345	1,269	4
S06	Intracranial injury	768,748	12,993	1.69	1,310	262	5	6,046	1,209	5
C22	Liver and intrahepatic bile duct cancer	125,268	15,904	12.70	1,290	258	6	4,769	954	6
A41	Sepsis	59,119	14,752	24.95	1,004	201	7	4,271	854	7
C25	Pancreatic cancer	78,966	8,391	10.63	930	186	8	3,247	649	10
I63	Cerebral infarction	332,018	10,398	3.13	739	148	9	2,771	554	13
C16	Stomach cancer	81,020	7,951	9.81	682	136	10	2,703	541	14
R57	Shock	31,028	10,337	33.32	602	120	11	3,468	694	8
J69	Pneumonitis due to solids and liquids	59,604	10,614	17.81	564	113	12	3,152	630	11
I21	Acute myocardial infarction	150,783	10,661	7.07	561	112	13	3,432	686	9
I61	Intracerebral haemorrhage	77,166	10,501	13.61	519	104	14	2,873	575	12
I50	Heart failure	132,791	8,896	6.70	509	102	15	2,668	534	15
J15	Bacterial pneumonia	47,671	5,371	11.27	471	94	16	2,044	409	17
N17	Acute kidney injury	74,364	6,353	8.54	454	91	17	1,831	366	19
C18	Colon cancer	71,652	5,566	7.77	436	87	18	2,401	480	16
R99	Other ill-defined and unspecified causes of mortality	27,708	26,557	95.85	280	56	19	1,674	335	20
I60	Subarachnoid haemorrhage	41,650	6,344	15.23	270	54	20	1,846	369	18

Note: This descriptive analysis used crude mortality rates and the full source population, including records with unknown age. Extended sepsis is excluded; A41, J15, J18, and J69 are retained separately. Short- and mid-term ranks were recalculated independently across these 20 conditions. The component results must not be added to the extended-sepsis composite. Cumulative values and ranks use unrounded estimates.

Supplementary Table S2. Annual and five-year cumulative crude short-term treatable-death estimates and rankings for the 17 primary-analysis condition groups, 2018–2022

Rank	Code or criterion	Study condition	2018	2019	2020	2021	2022	5-year cumulative	Mean annual
1	A41 + J15 + J18 + J69	Extended sepsis	661	839	520	481	593	3,094	618.7
2	C34	Bronchus and lung cancer	494	468	486	424	374	2,247	449.3
3	I46	Cardiac arrest	301	379	396	319	274	1,669	333.8
4	ICISS <0.9	Severe trauma	317	338	257	297	299	1,508	301.6
5	S06	Intracranial injury	261	275	258	268	247	1,309	261.9
6	C22	Liver and intrahepatic bile duct cancer	279	313	254	278	166	1,290	258.0
7	C25	Pancreatic cancer	171	221	184	171	183	930	186.0
8	I63	Cerebral infarction	137	136	139	155	172	739	147.8
9	C16	Stomach cancer	142	154	132	167	87	682	136.3
10	R57	Shock	101	132	112	112	146	602	120.5
11	I21	Acute myocardial infarction	97	154	79	110	122	561	112.2
12	I61	Intracerebral haemorrhage	122	92	86	106	114	519	103.9
13	I50	Heart failure	93	100	97	113	105	509	101.8
14	N17	Acute kidney injury	92	77	91	112	83	454	90.8
15	C18	Colon cancer	92	91	61	90	102	436	87.2
16	R99	Other ill-defined and unspecified causes of mortality	79	71	42	41	46	280	56.0
17	I60	Subarachnoid haemorrhage	52	63	69	48	38	270	54.1

Note: Short-term estimates represent deaths above the corresponding crude national mortality rate within the same condition and year. Crude estimates were calculated without age or sex adjustment using the same analytic population as the age- and sex-adjusted analyses. Ranks were based on full-precision five-year cumulative estimates; annual and cumulative estimates are displayed as whole deaths and mean annual estimates to one decimal place. ICD-10, International Classification of Diseases, 10th Revision; ICISS, International Classification of Diseases Injury Severity Score.

Supplementary Table S3. Annual and five-year cumulative crude mid-term treatable-death estimates and rankings for the 17 primary-analysis condition groups, 2018–2022

Rank	Code or criterion	Study condition	2018	2019	2020	2021	2022	5-year cumulative	Mean annual
1	I46	Cardiac arrest	4,162	4,730	5,171	4,401	3,537	22,002	4400.3
2	A41 + J15 + J18 + J69	Extended sepsis	1,840	1,838	4,438	4,307	3,706	16,129	3225.8
3	C34	Bronchus and lung cancer	1,013	1,689	1,061	1,461	1,349	6,573	1314.5
4	ICISS <0.9	Severe trauma	1,403	1,720	876	1,302	1,041	6,342	1268.4
5	S06	Intracranial injury	873	1,367	1,344	1,207	1,255	6,046	1209.1
6	C22	Liver and intrahepatic bile duct cancer	793	805	1,057	1,296	818	4,769	953.8
7	R57	Shock	547	824	668	683	746	3,468	693.6
8	I21	Acute myocardial infarction	578	699	399	1,029	727	3,432	686.4
9	C25	Pancreatic cancer	535	802	708	652	550	3,247	649.3
10	I61	Intracerebral haemorrhage	695	447	744	455	532	2,873	574.6
11	I63	Cerebral infarction	530	621	485	492	642	2,771	554.2
12	C16	Stomach cancer	353	586	691	669	404	2,703	540.6
13	I50	Heart failure	629	600	488	443	508	2,668	533.7
14	C18	Colon cancer	256	503	303	553	785	2,401	480.1
15	I60	Subarachnoid haemorrhage	312	339	352	310	533	1,846	369.2
16	N17	Acute kidney injury	430	453	355	375	217	1,831	366.2
17	R99	Other ill-defined and unspecified causes of mortality	585	246	286	212	346	1,675	335.0

Note: Mid-term estimates use the lowest crude regional mortality rate observed within the same condition and year as the target. Crude estimates were calculated without age or sex adjustment using the same analytic population as the age- and sex-adjusted analyses. Ranks were based on full-precision five-year cumulative estimates; annual and cumulative estimates are displayed as whole deaths and mean annual estimates to one decimal place. ICD-10, International Classification of Diseases, 10th Revision; ICISS, International Classification of Diseases Injury Severity Score.

Supplementary Table S4. Recurrent descriptive and CI-based regional elevations in age- and sex-adjusted standardized mortality ratios, 2018–2022

Code or criterion	Study condition	Regions with recurrent descriptive elevation, SMR > 1, n/16	Regions with recurrent CI-based elevation, 95% CI lower limit > 1, n/16
A41 + J15 + J18 + J69	Extended sepsis	10	3
C16	Stomach cancer	9	3
C18	Colon cancer	7	2
C22	Liver and intrahepatic bile duct cancer	12	4
C25	Pancreatic cancer	11	6
C34	Bronchus and lung cancer	11	7
I21	Acute myocardial infarction	8	2
I46	Cardiac arrest	5	1
I50	Heart failure	8	1
I60	Subarachnoid haemorrhage	7	0
I61	Intracerebral haemorrhage	9	1
I63	Cerebral infarction	8	2
ICISS <0.9	Severe trauma	9	4
N17	Acute kidney injury	10	1
R57	Shock	6	1
R99	Other ill-defined and unspecified causes of mortality	9	0
S06	Intracranial injury	7	3

Note: SMRs were adjusted simultaneously for sex and 10-year age groups using indirect standardization. Recurrent descriptive elevation and recurrent CI-based elevation were defined as meeting the corresponding criterion in at least 3 of the 5 study years. SMR > 1 denotes descriptive elevation; a 95% CI lower limit > 1 denotes CI-based elevation using the exact Poisson confidence interval. CI, confidence interval; SMR, standardized mortality ratio.

Supplementary Table S5. Sensitivity analyses of five-year cumulative age- and sex-standardized mid-term treatable-death estimates using alternative regional benchmarks, 2018–2022

Panel A. Comparison of single-lowest, annually selected pooled three-region, and five-year pooled three-region benchmarks

Study condition	Primary analysis<br>Annual lowest regional SMR	Sensitivity analysis<br>Annual three-region pooled SMR	Sensitivity analysis<br>Five-year three-region pooled SMR
Cardiac arrest (I46)	21,547 (1)	11,047 (2)	11,294 (2)
Extended sepsis (A41 + J15 + J18 + J69)	19,340 (2)	13,035 (1)	13,314 (1)
Severe trauma (ICISS <0.9)	7,010 (3)	4,972 (3)	4,208 (3)
Bronchus and lung cancer (C34)	6,715 (4)	4,353 (4)	3,811 (4)
Intracranial injury (S06)	5,240 (5)	3,496 (5)	2,919 (6)
Liver and intrahepatic bile duct cancer (C22)	4,710 (6)	3,004 (6)	2,929 (5)
Acute myocardial infarction (I21)	3,957 (7)	2,902 (7)	2,756 (7)
Shock (R57)	3,570 (8)	2,715 (8)	2,552 (8)
Pancreatic cancer (C25)	3,296 (9)	2,085 (11)	1,905 (10)
Intracerebral haemorrhage (I61)	2,994 (10)	2,251 (10)	2,023 (9)
Cerebral infarction (I63)	2,909 (11)	2,280 (9)	1,825 (11)
Heart failure (I50)	2,825 (12)	1,668 (13)	1,236 (13)
Stomach cancer (C16)	2,574 (13)	1,702 (12)	1,333 (12)
Colon cancer (C18)	2,366 (14)	1,153 (16)	844 (17)
Subarachnoid haemorrhage (I60)	1,755 (15)	1,252 (14)	976 (16)
Acute kidney injury (N17)	1,677 (16)	1,159 (15)	1,103 (14)
Other ill-defined and unspecified causes of mortality (R99)	1,662 (17)	1,092 (17)	1,000 (15)

Note: Values are five-year cumulative estimates, with ranks in parentheses. The primary benchmark was the lowest regional SMR for each condition and year. The annual three-region benchmark pooled observed and expected deaths across the three regions with the lowest annual SMRs. The five-year benchmark pooled observed and expected deaths across the three regions with the lowest five-year cumulative SMRs and was applied unchanged across all years. Each pooled benchmark was the sum of observed deaths divided by the sum of expected deaths. Regional estimates were truncated at zero within each year before summation. Expected deaths used the original condition- and year-specific age and sex standardization. Estimates are rounded to whole numbers; ranks were calculated before rounding. SMR, standardized mortality ratio; ICISS, International Classification of Diseases Injury Severity Score.

Supplementary Table S5 (continued)

Panel B. Sensitivity analysis using a pooled benchmark based on the three regions with the lowest SMRs

Code or criterion	Study condition	Primary Mid-term TD (single lowest region)	Sensitivity Mid-term TD (pooled lowest 3 regions)	Difference	Change, %	Primary rank	Sensitivity rank
I46	Cardiac arrest	21,547.0	11,047.0	-10,499.9	-48.7	1	2
A41 + J15 + J18 + J69	Extended sepsis	19,340.3	13,034.8	-6,305.5	-32.6	2	1
ICISS <0.9	Severe trauma	7,010.5	4,972.1	-2,038.3	-29.1	3	3
C34	Bronchus and lung cancer	6,715.5	4,353.0	-2,362.5	-35.2	4	4
S06	Intracranial injury	5,239.7	3,496.1	-1,743.6	-33.3	5	5
C22	Liver and intrahepatic bile duct cancer	4,710.3	3,003.6	-1,706.7	-36.2	6	6
I21	Acute myocardial infarction	3,956.6	2,902.4	-1,054.2	-26.6	7	7
R57	Shock	3,569.7	2,715.5	-854.2	-23.9	8	8
C25	Pancreatic cancer	3,295.7	2,084.9	-1,210.8	-36.7	9	11
I61	Intracerebral haemorrhage	2,994.0	2,251.3	-742.8	-24.8	10	10
I63	Cerebral infarction	2,909.0	2,280.0	-629.0	-21.6	11	9
I50	Heart failure	2,825.3	1,667.9	-1,157.4	-41.0	12	13
C16	Stomach cancer	2,574.3	1,701.5	-872.8	-33.9	13	12
C18	Colon cancer	2,366.3	1,152.8	-1,213.5	-51.3	14	16
I60	Subarachnoid haemorrhage	1,755.2	1,252.5	-502.7	-28.6	15	14
N17	Acute kidney injury	1,677.0	1,158.9	-518.1	-30.9	16	15
R99	Other ill-defined and unspecified causes of mortality	1,662.2	1,092.3	-570.0	-34.3	17	17

Note. The original 17 primary-analysis condition groups, including extended sepsis, were retained unchanged; only the Mid-term benchmark definition was modified. The pooled benchmark SMR was calculated as ΣO/ΣE across the three lowest-SMR regions for each condition-year. Top-5 overlap represents the number of conditions common to the five highest-ranked conditions under the primary and sensitivity benchmarks, regardless of rank order. Difference = sensitivity Mid-term TD − primary Mid-term TD; Change (%) = Difference / primary Mid-term TD × 100. The selected lowest-3 regions and their O, E, and SMR values are provided in the accompanying Supplementary Analysis Data workbook (sheet "S5 L3 Calculation"). TD, treatable death; O, observed deaths; E, expected deaths; SMR, standardized mortality ratio.

For the annually selected pooled lowest-three benchmark, rank concordance with the primary single-lowest benchmark across the 17 condition groups was high (Spearman $\rho=0.978$, $p<0.001$), and Top-5 overlap was 5/5. These statistics refer to the annually selected benchmark, not to the five-year pooled benchmark.

Supplementary Table S6. Sensitivity analysis after disaggregating extended sepsis into its four component conditions

Panel A. Five-year Treatable Death estimates and ranks for the 20-condition sensitivity analysis

Condition	Study condition	Short-term TD	Mid-term TD (single lowest regional SMR)	Pooled lowest-3 Mid-term TD	Short rank	Mid-term rank (single lowest regional SMR)	Pooled-3 rank
I46	Cardiac arrest	1,661.8	21,547.0	11,047.0	3	1	1
J18	Pneumonia, unspecified organism	1,990.0	12,955.2	9,514.0	1	2	2
ICISS <0.9	Severe trauma	1,537.9	7,010.5	4,972.1	4	3	3
C34	Bronchus and lung cancer	1,984.0	6,715.5	4,353.0	2	4	4
A41	Sepsis	892.4	5,316.2	3,516.0	7	5	5
S06	Intracranial injury	1,084.1	5,239.7	3,496.1	6	6	6
C22	Liver and intrahepatic bile duct cancer	1,225.7	4,710.3	3,003.6	5	7	7
I21	Acute myocardial infarction	598.0	3,956.6	2,902.4	12	8	8
R57	Shock	638.2	3,569.7	2,715.5	10	9	9
C25	Pancreatic cancer	868.8	3,295.7	2,084.9	8	10	13
J69	Pneumonitis due to solids and liquids	578.9	3,099.9	2,379.0	13	11	10
I61	Intracerebral haemorrhage	524.9	2,994.0	2,251.3	14	12	12
I63	Cerebral infarction	657.5	2,909.0	2,280.0	9	13	11
I50	Heart failure	479.3	2,825.3	1,667.9	15	14	15
C16	Stomach cancer	630.9	2,574.3	1,701.5	11	15	14
C18	Colon cancer	416.1	2,366.3	1,152.8	17	16	19
J15	Bacterial pneumonia	359.9	2,140.5	1,424.2	18	17	16
I60	Subarachnoid haemorrhage	270.3	1,755.2	1,252.5	20	18	17
N17	Acute kidney injury	447.9	1,677.0	1,158.9	16	19	18
R99	Other ill-defined and unspecified causes of mortality	270.5	1,662.2	1,092.3	19	20	20

Extended sepsis was removed and replaced by A41, J15, J18, and J69; all other primary-analysis conditions were retained, yielding 20 condition groups. Ranks were calculated independently across the 20 condition groups. Estimates are age- and sex-adjusted and are shown to one decimal place; ranks were calculated before rounding. The pooled lowest-three benchmark was selected separately for each condition and year. TD, treatable death; SMR, standardized mortality ratio.

Supplementary Table S6 (continued)

Panel B. Frequency of annual regional SMR > 1, 2018–2022

Condition	Study condition	A	B	C	D	E	F	G	H	I	J	K	L	M	N	O	P
I46	Cardiac arrest	5	5	0	0	1	5	2	5	0	0	1	1	2	5	0	0
J18	Pneumonia, unspecified organism	4	3	5	0	5	5	5	0	2	0	1	4	0	5	0	1
ICISS <0.9	Severe trauma	2	4	5	0	5	5	3	0	5	4	5	0	2	4	0	2
C34	Bronchus and lung cancer	0	5	5	1	4	5	5	0	5	3	1	5	5	5	0	5
A41	Sepsis	0	4	3	0	4	1	4	4	1	5	5	4	5	2	0	3
S06	Intracranial injury	1	5	5	0	4	1	2	0	0	0	5	2	3	5	0	3
C22	Liver and intrahepatic bile duct cancer	3	5	5	1	3	5	5	0	5	4	1	3	4	3	0	5
I21	Acute myocardial infarction	3	4	1	0	3	5	3	3	0	4	5	2	1	0	0	0
R57	Shock	1	2	5	0	2	0	0	4	3	1	4	5	1	2	0	5
C25	Pancreatic cancer	3	5	5	2	5	5	5	0	5	0	1	4	4	4	0	5
J69	Pneumonitis due to solids and liquids	1	5	3	0	0	2	5	0	3	1	1	5	3	5	1	3
I61	Intracerebral haemorrhage	3	4	3	0	5	5	4	1	4	4	1	5	1	1	0	0
I63	Cerebral infarction	2	1	2	0	5	4	5	1	3	2	5	4	4	1	3	1
I50	Heart failure	4	0	2	0	5	4	5	0	5	1	3	5	0	1	5	1
C16	Stomach cancer	3	5	5	1	4	5	5	0	5	2	1	2	5	0	0	5
C18	Colon cancer	1	4	5	2	2	5	5	0	5	1	2	2	3	0	1	5
J15	Bacterial pneumonia	1	5	0	1	2	3	4	4	5	3	5	3	2	1	0	4
I60	Subarachnoid haemorrhage	2	5	3	0	2	5	1	2	0	3	5	5	1	4	0	1
N17	Acute kidney injury	1	4	4	4	5	2	5	0	4	1	2	4	1	3	5	3
R99	Other ill-defined and unspecified causes of mortality	5	5	5	3	2	1	0	5	5	0	5	5	1	5	0	0

Cell value = number of years with annual regional Age×Sex 10-year adjusted SMR > 1 (0–5). Shaded values indicate recurrent elevation (≥3 of 5 years). Region labels A–P are anonymized consistently with Supplementary Figure S1; no crosswalk is provided. CI, confidence interval; SMR, standardized mortality ratio.

Supplementary Table S6 (continued)

Panel C. Frequency of annual regional Exact Poisson 95% CI lower bound > 1, 2018–2022

Condition	Study condition	A	B	C	D	E	F	G	H	I	J	K	L	M	N	O	P
I46	Cardiac arrest	2	0	0	0	0	0	0	1	0	0	0	0	0	3	0	0
J18	Pneumonia, unspecified organism	1	1	0	0	5	4	4	0	0	0	0	1	0	4	0	1
ICISS <0.9	Severe trauma	0	1	5	0	5	3	1	0	0	0	2	0	0	3	0	0
C34	Bronchus and lung cancer	0	4	4	0	4	4	4	0	5	0	1	1	0	2	0	5
A41	Sepsis	0	1	1	0	0	0	1	2	0	1	2	1	4	0	0	3
S06	Intracranial injury	0	4	5	0	1	0	0	0	0	0	2	0	0	5	0	1
C22	Liver and intrahepatic bile duct cancer	0	4	2	0	1	5	5	0	2	0	1	1	1	0	0	4
I21	Acute myocardial infarction	0	2	0	0	0	3	0	1	0	0	4	0	0	0	0	0
R57	Shock	0	0	5	0	0	0	0	1	0	0	1	2	0	0	0	1
C25	Pancreatic cancer	1	4	2	0	3	5	4	0	3	0	0	1	1	1	0	4
J69	Pneumonitis due to solids and liquids	0	4	0	0	0	0	3	0	0	0	0	0	0	3	0	0
I61	Intracerebral haemorrhage	0	0	0	0	4	0	1	1	0	1	0	1	0	0	0	0
I63	Cerebral infarction	0	0	0	0	5	1	4	0	1	0	1	1	0	0	0	0
I50	Heart failure	0	0	0	0	1	1	4	0	1	0	0	2	0	0	1	0
C16	Stomach cancer	0	4	2	1	2	1	3	0	0	0	1	0	0	0	0	3
C18	Colon cancer	0	2	0	0	0	2	0	0	4	0	0	1	0	0	0	3
J15	Bacterial pneumonia	0	2	0	0	1	0	0	0	0	0	1	0	0	0	0	0
I60	Subarachnoid haemorrhage	0	1	0	0	0	2	0	0	0	0	0	0	0	0	0	0
N17	Acute kidney injury	0	1	0	0	1	0	5	0	0	0	0	1	0	0	2	0
R99	Other ill-defined and unspecified causes of mortality	0	0	0	0	0	0	0	0	0	0	0	0	0	0	0	0

Cell value = number of years in which the lower bound of the Exact Poisson 95% CI for SMR exceeded 1 (0–5). Shaded values indicate recurrent statistically significant elevation (≥3 of 5 years). Region labels A–P are anonymized consistently with Supplementary Figure S1; no crosswalk is provided. CI, confidence interval; SMR, standardized mortality ratio.

Supplementary Table S6 (continued)

Panel D. Number of regions showing recurrent elevation (≥3 of 5 years)

Condition	Study condition	Regions with SMR >1 in ≥3 of 5 years	Regions with 95% CI lower bound >1 in ≥3 of 5 years
I46	Cardiac arrest	5	1
J18	Pneumonia, unspecified organism	8	4
ICISS <0.9	Severe trauma	9	4
C34	Bronchus and lung cancer	11	7
A41	Sepsis	10	2
S06	Intracranial injury	7	3
C22	Liver and intrahepatic bile duct cancer	12	4
I21	Acute myocardial infarction	8	2
R57	Shock	6	1
C25	Pancreatic cancer	11	6
J69	Pneumonitis due to solids and liquids	8	3
I61	Intracerebral haemorrhage	9	1
I63	Cerebral infarction	8	2
I50	Heart failure	8	1
C16	Stomach cancer	9	3
C18	Colon cancer	7	2
J15	Bacterial pneumonia	9	0
I60	Subarachnoid haemorrhage	7	0
N17	Acute kidney injury	10	1
R99	Other ill-defined and unspecified causes of mortality	9	0

Treatable-death estimates are operational policy-scenario estimates and should not be interpreted as estimates of causally preventable mortality.

CI, confidence interval; SMR, standardized mortality ratio.

Supplementary Figure S1. Frequency of elevated age- and sex-adjusted SMRs by condition and anonymized region, 2018–2022.

Panel A shows the number of study years (0–5) in which each region had an SMR > 1. Panel B shows the number of study years (0–5) in which the lower limit of the exact Poisson 95% CI was > 1. SMRs were adjusted simultaneously for sex and 10-year age groups using indirect standardization. Region labels are anonymized and no crosswalk is provided. CI, confidence interval; SMR, standardized mortality ratio.

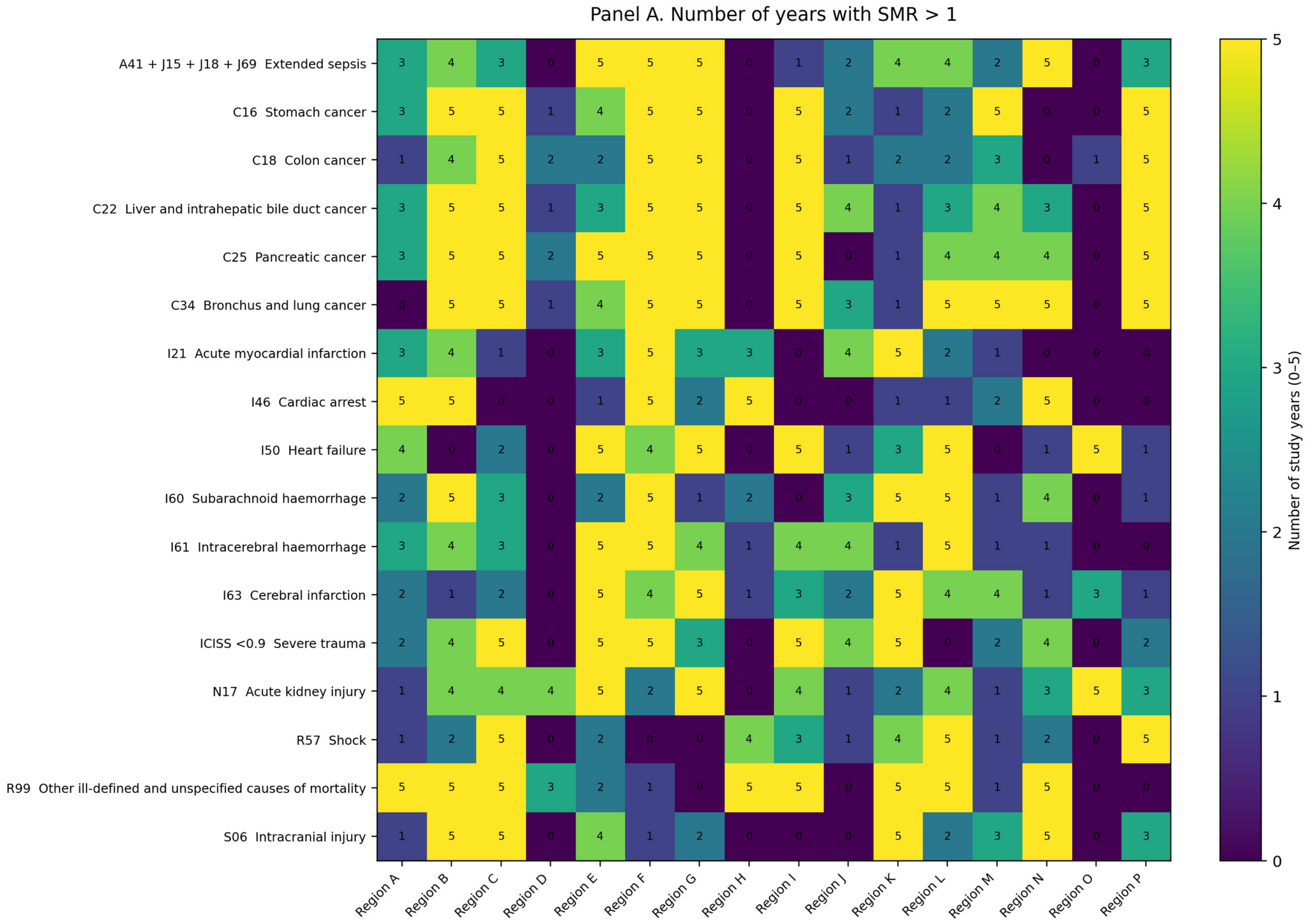

Supplementary Figure S1 (continued)

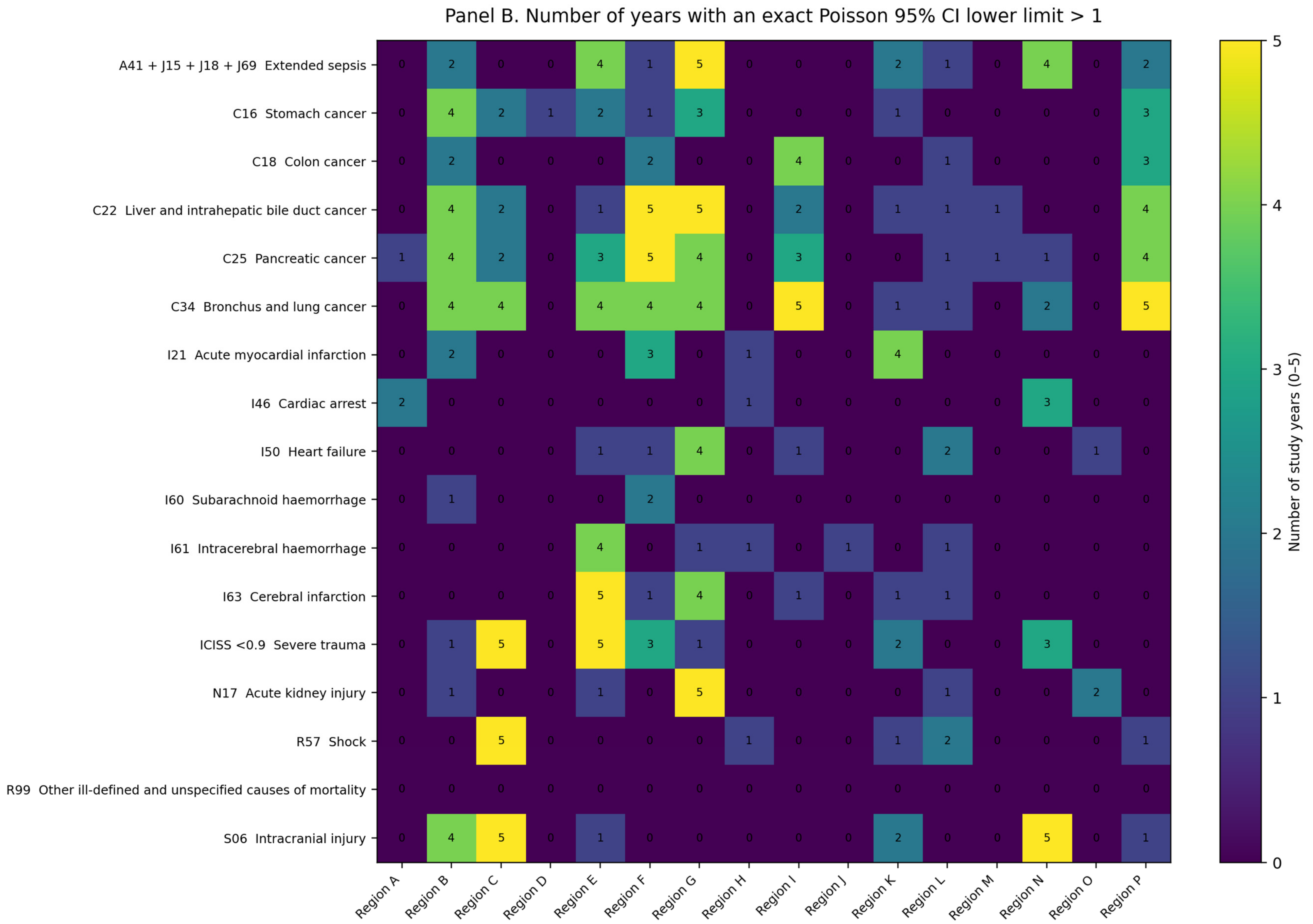

Supplement: Supplementary file 1 [file jcm-15-07848-s001.zip › jcm-4579899-supplementary.pdf]
